# Breath-hold calibrated fMRI mapping of absolute cerebral metabolic rate of oxygen metabolism (CMRO_2_): an assessment of the accuracy and repeatability in a healthy adult population

**DOI:** 10.1162/imag_a_00298

**Published:** 2024-09-23

**Authors:** Ian D. Driver, Antonio Maria Chiarelli, Hannah L. Chandler, Hannah Thomas, Svetla Manolova, Hanzhang Lu, Richard G. Wise, Michael Germuska

**Affiliations:** 1 Cardiff University Brain Research Imaging Centre (CUBRIC), School of Physics and Astronomy, Cardiff University, Cardiff, United Kingdom; 2 Cardiff University Brain Research Imaging Centre (CUBRIC), School of Psychology, Cardiff University, Cardiff, United Kingdom; 3 Department of Neuroscience, Imaging, and Clinical Sciences, University G. D'Annunzio of Chieti-Pescara, Chieti, Italy; 4 Institute for Advanced Biomedical Technologies (ITAB), University G. D'Annunzio of Chieti-Pescara, Chieti, Italy; 5 The Russell H. Morgan Department of Radiology & Radiological Science, Johns Hopkins University School of Medicine, Baltimore, MD, United States; 6 Department of Biomedical Engineering, Johns Hopkins University School of Medicine, Baltimore, MD, United States; 7 F.M. Kirby Research Center for Functional Brain Imaging, Kennedy Krieger Research Institute, Baltimore, MD, United States; 8 Department of Radiology, University of California Davis Medical Center, Sacramento, California

**Keywords:** Cerebral oxygen metabolism, oxygen extraction fraction, calibrated BOLD, oxygen exchange, breath-hold, fMRI

## Abstract

We previously introduced a calibrated fMRI framework that utilises respiratory modulation with only a single gas (CO_2_) to map the grey matter (GM) cerebral metabolic rate of oxygen consumption (CMRO_2_). The method decouples and estimates the cerebral blood volume (CBV) and the oxygen extraction fraction (OEF) from a single measure of the maximum BOLD modulation. The method links the two parameters of interest with a model of oxygen diffusion from capillaries to mitochondria which incorporates the cerebral blood flow (CBF). Here we apply this framework to gas-free breath hold calibrated fMRI (bhc-fMRI), where simultaneous BOLD and ASL acquisitions are combined with modulation of arterial CO_2_ through repeated breath-holding. The accuracy and repeatability of the method is assessed in 33 healthy volunteers at rest and during continuous visual stimulation. Average GM OEF estimated from bhc-fMRI was 0.37 ± 0.04 indicating a small bias of 0.04 (with limits of agreement from -0.11 to 0.12) compared to the whole brain OEF of 0.32 ± 0.07 estimated from sagittal sinus using T2 Relaxation Under Spin Tagging (TRUST). The within session repeatability of GM estimates were moderate to good for OEF, with ICC = 0.75 (0.56-0.87) and good to excellent for CMRO_2_, with ICC = 0.88 (0.74-0.94). An ROI analysis in the visual cortex found an average CBF increase of 16%, a CMRO_2_ increase of 12%, and an OEF decrease of 3% during the visual stimulation. The bhc-fMRI measurement of CMRO_2_ is simple to implement, has comparable accuracy and repeatability to existing gas-based methods and is sensitive to modulations in metabolism during functional hyperaemia.

## 1 Introduction

A continuous supply of oxygen to the brain is essential for life and restriction of this supply can have significant consequences for individuals (Safar, 1988). Furthermore, alterations in cerebral oxygen metabolism are associated with inflammation (Paling et al., 2011), neurodegeneration (Robb et al., 2022), tumour (Paech et al., 2020), and traumatic brain injury (Ragan et al., 2013). Thus, convenient methods for mapping cerebral oxygen metabolism could provide valuable information for patient care and aid understanding of disease aetiology, progression and treatment response.

Magnetic resonance imaging (MRI) is sensitive to the presence of deoxyhaemoglobin in the cerebral vasculature, permitting the mapping of oxygen extraction fraction (OEF) and the cerebral rate oxygen metabolism (CMRO_2_). Several MRI methods that exploit this deoxyhaemoglobin sensitivity have been proposed, each with their own advantages and limitations (Biondetti et al., 2023; Chen et al., 2022; Jiang & Lu, 2022). For example, oxygen extraction fraction can be mapped by measuring the effect of deoxyhaemoglobin on transverse relaxation or local susceptibility (An & Lin, 2000; Cherukara et al., 2019; Cho et al., 2021; He & Yablonskiy, 2007; Lee & Wehrli, 2022; Ulrich & Yablonskiy, 2016). However, these methods are limited by being unable to distinguish local field shifts caused by haemoglobin from those originating from other sources, such as non-haem iron or myelin. Further challenges with these methods lie in disentangling deoxygenated blood volume from oxygen extraction fraction and their susceptibility to bias from field inhomogeneities (Christen et al., 2014). Another group of MRI methods, termed “calibrated” functional MRI (fMRI), are based on the concept of estimating the maximum blood oxygen level dependant (BOLD) signal modulation, i.e. the BOLD signal increase that would be obtained with a complete removal of the deoxyhemoglobin from the voxel, exploiting functional signals acquired during isometabolic gas challenges. Compared to other approaches, these calibrated fMRI methods have an advantage in that they uncouple the signal arising from deoxyhaemoglobin from other brain sources of susceptibility. However, dual-calibrated gas methods (Bulte et al., 2012; Gauthier et al., 2012; Wise et al., 2013) are cumbersome to implement and are sensitive to noise. We recently introduced a single gas calibration framework that incorporates a model of oxygen transport to simplify the experimental requirements and reduce the noise sensitivity of the method (Chiarelli et al., 2022). Modelling studies and experimental comparison with the dual-calibrated approach demonstrated similar uncertainty in estimates of OEF, and good agreement between methods when using exogenous CO_2_ for respiratory modulation.

The primary cerebrovascular response to breath-holding (on expiration) is an arterial CO_2_ induced vasodilation and associated increase in cerebral blood flow (CBF). However, there is also a small reduction in arterial oxygen saturation (Sasse et al., 1996), which dampens the BOLD response compared to the exogenous CO_2_ modulation previously employed. Although the physiological response to breath-holding is more complicated than that arising from the administration of CO_2_, the experimental procedure is greatly simplified and more comfortable for many subjects. Therefore, we chose to investigate if repeated breath-holding could be used for the quantification of GM OEF during rest and during continuous visual stimulation, where local changes in perfusion are expected to outstrip the increase in CMRO_2_, leading to a decrease in OEF. To validate the accuracy of the breath-hold calibrated fMRI (bhc-fMRI) method against an independent and robust measurement of OEF, average GM estimates of OEF were compared to whole brain measurements acquired with the T_2_-Relaxation-Under-Spin-Tagging (TRUST) method (Lu & Ge, 2008), a method which, itself, has been validated against a gold standard oximetry method (Lu et al., 2012).

## 2 Methods

### 2.1 Biophysical Modelling

Here we summarize the biophysical model used for parameter estimation. For a detailed description of the model please refer to Chiarelli & Germuska (Chiarelli et al., 2022).

Following the general BOLD calibration modelling of (Gauthier & Hoge, 2013) the maximum BOLD signal (M) for a breath-holding stimulus can be expressed as:

(1) $$M_{calib\;}=\frac{\text{Δ}BOLD}{BOLD_{0}}/\left\{1-{\left(\frac{CBF}{CBF_{0}}\right)}^{\alpha }\cdot {\left(\frac{1-\frac{C_{a}O_{2}}{\varphi \cdot [Hb]}\cdot \left(1-\frac{OEF_{0}\cdot CBF_{0}\cdot C_{a}O_{2,0}}{CBFC_{a}O_{2}}\right)}{1-\frac{CaO_{2}}{\varphi \cdot [Hb]}\cdot \left(1-OEF_{0}\right)}\right)}^{\beta }\right\}$$

The subscript 0 depicts the baseline parameter value, M_calib_ is the maximum BOLD signal (as estimated by the general BOLD calibration model), CBF is cerebral blood flow (mL/100g/min), CaO_2_ is the arterial oxygen content in blood (mL/dL), *φ* is the oxygen binding capacity of haemoglobin (1.34 mL/g), [Hb] is the concentration of haemoglobin in blood (g/dL), OEF is the oxygen extraction fraction, α (0.2 (Chen & Pike, 2010) ) is the Grubb exponent relating fractional change in cerebral blood flow to the change in deoxyhaemoglobin-weighted blood volume, and β (1.3 at 3T) is a field strength and vessel geometry dependent exponent.

Alternatively, as we have shown previously (Chiarelli et al., 2022) and as demonstrated in the supplementary materials (S1), M can be derived with reference to a flow diffusion model of oxygen exchange from the capillary bed:

(2) $$M_{diffusion\;}=TE\cdot \frac{A\cdot \rho }{K}\cdot \frac{OEF_{0}\cdot CBF_{0}\cdot C_{a}O_{2,0}\cdot {(\left(1-\frac{C_{a}O_{2,0}}{\varphi \cdot [Hb]}\cdot \left(1-OEF_{0}\right))\cdot [Hb\right])}^{\beta }}{\left(P_{50}\cdot \sqrt[{h}]{\frac{2}{OEF_{0}}-1}-P_{m}O_{2,0}\right)}$$

where TE is the echo time of the BOLD acquisition (s), A is a field strength and vessel geometry dependent constant (s^-1^g^-β^dL^β^), ρ is the ratio of the BOLD sensitive blood volume to the capillary blood volume, K is the effective permeability to oxygen of the capillary endothelium and brain tissue (μmol/mmHg/mL/min), P_50_ is the oxygen partial pressure when half the haemoglobin in blood is saturated (26 mmHg), h is the Hill constant for cooperative binding of oxygen to haemoglobin (2.84), and PmO_2_ is the partial pressure of oxygen at the mitochondria (≈ 0 mmHg).

We assigned a value of 8.85 s^-1^g^-β^dL^β^ /(μmol/mmHg/mL/min) to$\frac{A\cdot \rho }{K}$, matching our previously establish in-vivo measurement when PmO_2_ is fixed to 0 mmHg (Chiarelli et al., 2022).

In both equations 1 and 2 the only unknown parameters, that are neither measured nor assigned a value from expected physiology, are the maximum BOLD signal (M) and the resting oxygen extraction fraction (OEF_0_). Therefore, OEF_0_ can be found by searching for the value that minimises the difference between the two estimates of M (M_calib_ and M_diffusion_).

### 2.2 Data Acquisition

Thirty-five healthy volunteers (16 female, mean age 24.5 ± 6 years) were recruited. The study was conducted in accordance with the Declaration of Helsinki and was approved by the Cardiff University, School of Psychology Ethics Committee. Written informed consent was obtained from each participant. Data were acquired using a Siemens MAGNETOM Prisma (Siemens Healthcare GmbH, Erlangen) 3T MRI scanner with a 32-channel receive only head coil (Siemens Healthcare GmbH, Erlangen). Data from one participant were discarded due to the presence of a vascular abnormality. Data from a second participant were discarded as there was no signal from the anterior elements of the head coil. Therefore, data from 33 participants were analysed as follows.

An in-house dual-excitation (Schmithorst et al., 2014) (DEXI) pCASL sequence with two inversion pulses for background suppression was used to collect simultaneous BOLD and ASL data during repeated breath-holding at rest and during continual visual stimulation. The labelling duration and the Post Label Delay (PLD) were both set to 1.5 s, GRAPPA acceleration (factor = 3) was used with TE_1_ = 10 ms and TE_2_ = 30 ms. A TR of 4.4 s was used to acquire 15 2D EPI slices, in-plane resolution 3.4 mm x 3.4 mm and slice thickness 6 mm (33% slice gap). The sequence timings are shown in Figure 3 of (Germuska et al., 2019), with BOLD data acquired 1 s after ASL data.

**Figure 1:**
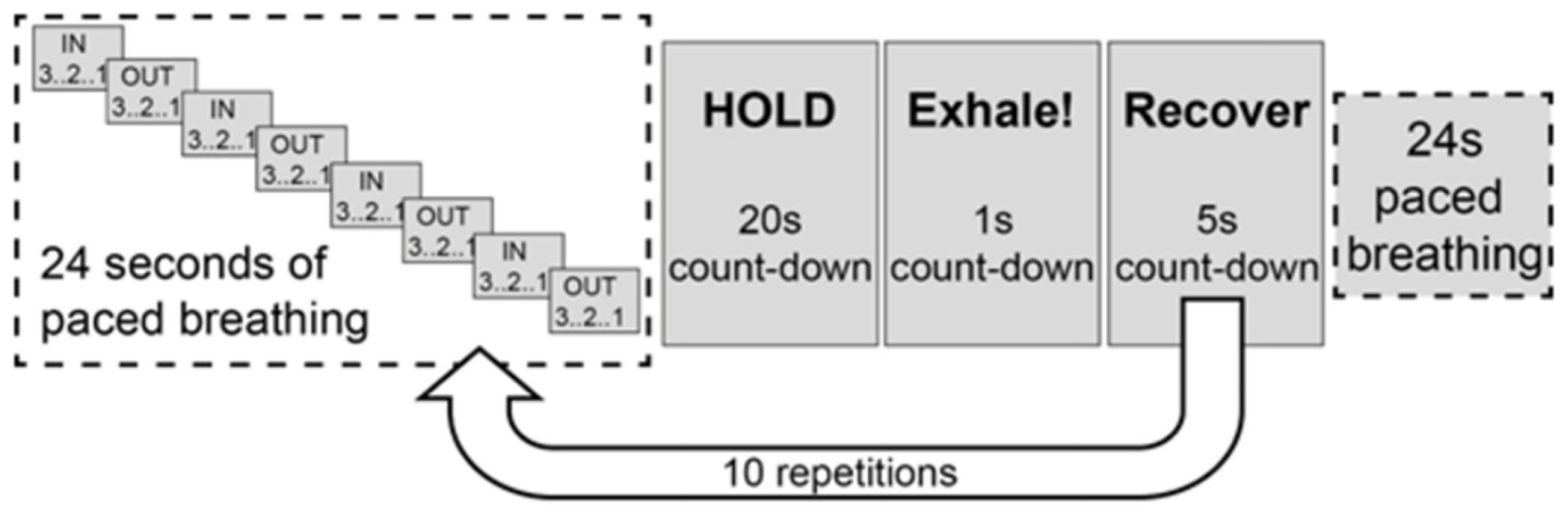
Schematic of the breath-holding paradigm used in experiments. The paradigm was repeated two times, with and without a visual stimulation consisting of an isoluminant reversing radial checkerboard.

The breath-holding protocol consisted of 10 breath-holds, each of 20 seconds duration, with 30 seconds of recovery in-between (Figure 1). As recommended previously (Bright & Murphy, 2013), for reliability of the breath-hold fMRI measurement, the breath-hold started at end-expiration and participants were instructed to breathe out at the end of each breath hold so that the arterial oxygen partial pressure could be estimated, see Figure 1 for experimental details. Participants were given the opportunity to run through a single trial breath-hold cycle whilst lying on the scanner bed, but before being moved into the bore. They were also asked to breathe through their nose during the breath-hold task to allow sampling through a nasal cannula. A respiratory bellows was used to record the movement of the abdomen during data collection and respiratory CO_2_ and O_2_ were recorded from the subject’s nasal cannula using a gas analyser (AEI Technologies, Pittsburgh, PA, USA). The total time for the breath-holding paradigm was 8 minutes 44 seconds.

The breath-holding protocol and resulting breath-hold calibration measurement were repeated in the same session, providing two separate measurements of OEF and CMRO_2_, approximately 30 minutes apart. During the first run, the background of the breath holding instructions displayed on the in-bore screen was grey. During the second run, termed visual experiment, the entire DEXI-pCASL acquisition was repeated with the breath holding instructions displayed in the centre of an isoluminant reversing radial checkerboard (reversal frequency of 8Hz). The centre portion of the screen remained grey, so the breath hold instructions (Figure 1) were clear to read. The reversing checkerboard was sustained throughout the breath-hold task. The visual presentation scripts for the breath-hold and breath-hold with visual stimulation tasks are available from *git.cardiff.ac.uk/cubric/wand/-/tree/main/code/BreathHold*.

A separate proton density weighted image was acquired prior to each DEXI-pCASL scan for quantitative ASL calibration with TR = 6 seconds and TE = 10 ms. All other parameters were set to match the DEXI-pCASL acquisition, excluding pre-saturation, pCASL tagging and background suppression inversion pulses, which were switched off.

A T_2_-Relaxation-Under-Spin-Tagging (TRUST) MRI sequence (Lu & Ge, 2008) was used to estimate global OEF from the sagittal sinus oxygen saturation. TR / TE = 3000 ms / 3.9 ms, in-plane resolution 3.4 mm x 3.4 mm, slice thickness = 5 mm, effective TEs= 0 ms, 40 ms, 80 ms, 160 ms, GRAPPA acceleration factor = 3, partial Fourier = 6/8, 24 acquisitions (3 repeats of 4 tag/control pairs). An inversion recovery sequence was acquired with the same prescription as the TRUST sequence through the sagittal sinus, ΔTR / TE = 150 ms / 22 ms, in-plane resolution 1.9 mm x 1.9 mm, slice thickness = 3 mm, GRAPPA acceleration factor = 2, partial Fourier = 7/8, 960 acquisitions (16 repeats of 60 measurements). The inversion recovery sequence was used to quantify the T_1_ of venous blood (Varela et al., 2011) for estimation of systemic [Hb] used in the calculation of OEF in both the breath-hold calibrated framework and the TRUST analysis.

A magnetisation-prepared rapid acquisition with gradient echo (MPRAGE) T_1_-weighted scan was acquired for registration and brain segmentation purposes (matrix 165 x 203 x 197, 1 mm isotropic resolution, TR/TE/TI = 2100/3.24/850 ms, flip angle 8°).

### 2.3 Data Analysis

#### 2.3.1 Global [Hb] and OEF_0_ estimation

The T_1_ of venous blood was estimated from non-linear least squares fitting to a mono-exponential signal model using the long TR approximation, *S* = *abs*(*a* + *b* · exp (−*TI*/*T*_1_)). To reduce possible contamination from blood water of non-venous origin at long recovery times, only the first 40 data points were used from each repetition of the inversion recovery acquisition. Automatic voxel selection was achieved in a two-step procedure. First the 5 most intense voxels in a ROI including the sagittal sinus were identified from the second time point of the inversion recovery acquisition, then the voxel with the smallest relative deviation from the mono-exponential model was chosen for analysis. The blood Hct was determined from the linear relationship with venous T_1_ previously reported at 3T (Lu et al., 2004), *T*_1_(*s*) = 1/(0.83 · *Hct* + 0.28). Hct was converted to [Hb], for analysis of the breath-hold calibrated data, via the empirical relationship reported by Kokholm (Kokholm, 1990), *Hct* = 0.0485 · [*Hb*](*mmol/L*) + 0.0083.

The T_2_ of blood was found by nonlinear least squares fitting of a mono-exponential equation to the TRUST difference data as a function of T_2_ and the effective echo time. The two most intense voxels from the TRUST difference data, from an ROI including the sagittal sinus, were selected for TRUST data analysis. The venous oxygen saturation (Y_v_) was found by inverting the relationship between blood T_2_, Hct, and Y_v_ reported by Lu et al (Lu et al., 2012). OEF was calculated as the fractional difference between arterial and venous oxygen saturation, *OEF* = (*Y*_*a*_ – *Y*_*v*_)/*Y*_*a*_, with the arterial saturation assumed to be 0.98.

#### 2.3.2 Breath-hold calibrated estimation of OEF

Voxelwise estimation of OEF_0_ follows the same method as the analysis previously presented for single gas calibrated estimation (Chiarelli et al., 2022). However, instead of a regressor produced from end-tidal traces of O_2_ or CO_2_, the CO_2_ regressor for breath-holding was inferred from the global ASL and BOLD data on a subject wise basis. Additionally, we used the group average PaO_2_ (oxygen partial pressure during breath-holding) and PaO2_0_ (oxygen partial pressure during room air breathing), 104 mmHg and 127 mmHg respectively, to calculate CaO_2_ and CaO_2,0_ via equations 3 and 4. This data-driven approach removes the reliance on individual physiological recordings acquired during MRI data acquisition, which are often of poor data quality (Zvolanek et al., 2023).

(3) $$SaO_{2}={\left(\left({\left(PaO_{2}^{3}+150\cdot PaO_{2}\right)}^{-1}\cdot 23400\right)+1\right)}^{-1}$$

(4) $$CaO_{2}=[Hb]\cdot \varphi \cdot SaO_{2}+PaO_{2}\cdot \varepsilon$$

**Figure 2:**
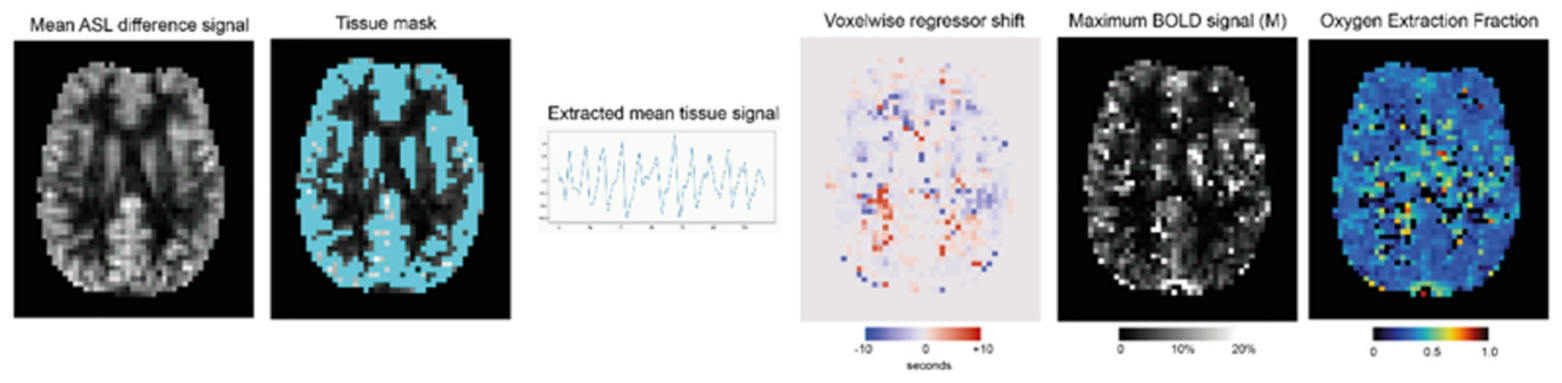
Processing steps for OEF estimation. 1) Calculation of mean ASL difference signal to create grey matter tissue mask. 2) Extraction of mean ASL and BOLD signal from tissue mask to create a physiological regressor. 3) Calculate voxelwise shift with respect to global regressor. 4) Simultaneous estimation of maximum BOLD signal and OEF_0_ that produces the best fit to the data.

SaO_2_ is the oxygen saturation of arterial haemoglobin, PaO_2_ is the arterial oxygen partial pressure and ε is the solubility coefficient of oxygen in blood (0.003 mL/dL/mmHg). The analysis code for estimating OEF_0_ and CMRO_2_ is publicly available (*10.5281/zenodo.10695877*), the main steps of the analysis are shown in Figure 2 and outlined below.

DEXI-pCASL data were separated into two time series, one for TE_1_ and one for TE_2_. Motion correction of each time series was applied using 6 degrees of freedom co-registration using MCFLIRT (Jenkinson et al., 2002). Perfusion-weighted and BOLD-weighted time series were derived from TE_1_ and TE_2_ time series via surround subtraction and surround averaging respectively. The perfusion and BOLD data were then spatially smoothed in 2D with a Gaussian filter (sigma = 0.5 pixels). Voxelwise timeseries were then high pass filtered with a FWHM cutoff of 100 seconds using the filter implementation from FSL (Jenkinson et al., 2012). Since baseline CBF is larger in the grey matter (GM) compared to WM, the mean perfusion map was used to estimate the GM partial volume, thus avoiding errors introduced by registration with anatomy. The mean perfusion signal was thresholded between the 85^th^ and 99^th^ percentiles to create a mask of voxels with a perfusion signal (GM mask).

The mean timeseries from ASL and BOLD voxels within the GM mask was used to create a physiological regressor that relates to the global breath-holding stimulus. A weighted average of the normalised mean BOLD and ASL timeseries was used to produce the regression vector. Empirically it was found that a ratio of 2:1 (BOLD:ASL) created a robust regressor for analysis. Cross-correlation between the regressor and concatenated BOLD and ASL data was used to estimate the voxelwise temporal shift relative to the global signal. Regression against BOLD and ASL timeseries was then used to determine CBF_0_, the fractional change in CBF, and the BOLD signal change. Finally, equations 1 and 2 were used to estimate M_calib_ and M_diffusion_ for 1000 OEF values between 0.001 and 1, in 0.001 steps. The OEF value that produced the minimum difference between M_calib_ and M_diffusion_ was kept as the solution for OEF_0_. CMRO_2_ was calculated via the Fick principle, as expressed in equation 5.

(5) $$CMRO_{2}=CaO_{2}\cdot OEF_{0}\cdot CBF_{0}$$

For comparison with the global TRUST measurement of OEF_0_, the mean value from the breath-hold calibrated analysis was calculated within the GM mask.

#### 2.3.3 Group analysis and statistics

The transformation to realign parameter maps to MNI152 space (Fonov et al., 2011) was calculated by concatenating two transformations: (i) CBF_0_ to MPRAGE and (ii) MPRAGE to MNI152. (i) CBF_0_ maps were coregistered to the GM partial volume estimate map of the MPRAGE using FSL FLIRT (Jenkinson et al., 2002) with a *normalised correlation* cost function and 6 degrees of freedom. (ii) The MPRAGE was coregistered to MNI152 space using FSL FNIRT (Jenkinson et al., 2012) with the *T1_2_MNI152_2mm* configuration settings, 10mm warp resolution and an initial FLIRT affine transformation calculated with a *correlation ratio* cost function and 12 degrees of freedom.

Parameter maps (CBF_0_, OEF_0_, CMRO_2_,_0_) were smoothed with a 6.88 mm FWHM Gaussian kernel (twice the in-plane resolution) before being realigned into MNI152 space. Two sets of parameter maps were acquired, one with the breath-hold task only and the other with breath-hold and visual stimulation, as detailed in section 2.2 Data Acquisition. Statistical parametric group maps testing the difference in parameter map between breath-hold only and breath-hold with visual stimulation conditions were formed using a two-sample paired t-test in FSL Randomise (Winkler et al., 2014) with 10000 permutations and threshold-free cluster enhancement (Smith & Nichols, 2009). Significant activation was determined as regions with a family-wise error (FWE)-corrected p_FWE_<0.05.

Further, a visual region of interest (ROI) was defined based on the CBF p_FWE_<0.05 region. Mean values of each parameter (in group space) were calculated over this CBF ROI for each condition and the ROI-average response to visual stimulation was taken as the difference between conditions of each parameter.

Normality was tested by the Shapiro-Wilk test. For t-tests, the paired difference was tested. For correlation and Bland-Altman analysis, each variable was tested separately. If either variable showed evidence of not being normally distributed (p<0.05), then a Spearman ρ correlation was reported, whilst median and 95% limits of agreement were displayed in the Bland-Altman plot.

## 3 Results

**Figure 3:**
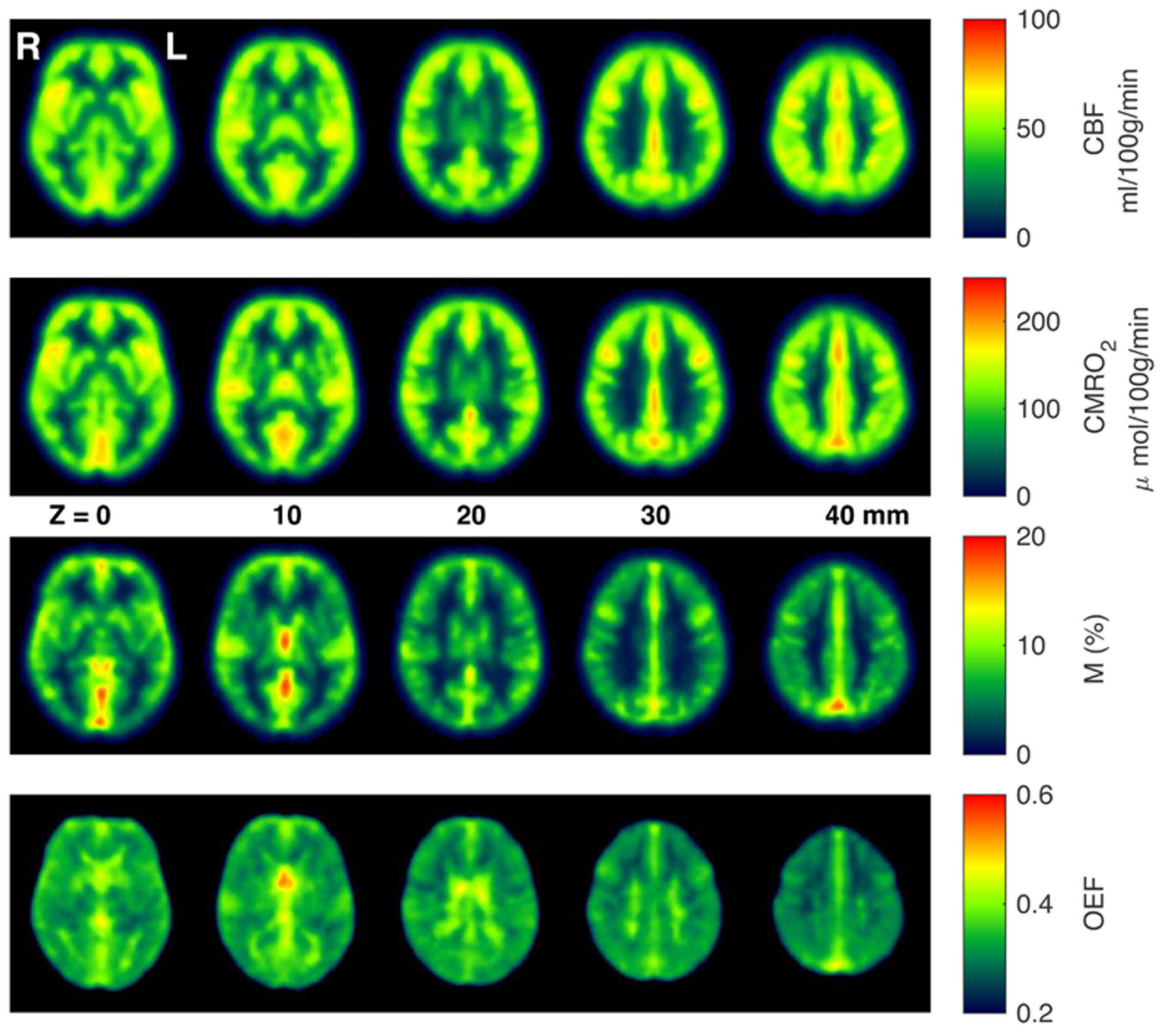
Example group averaged parameter maps from 33 subjects acquired during rest, after registration to MNI space.

**Figure 4:**
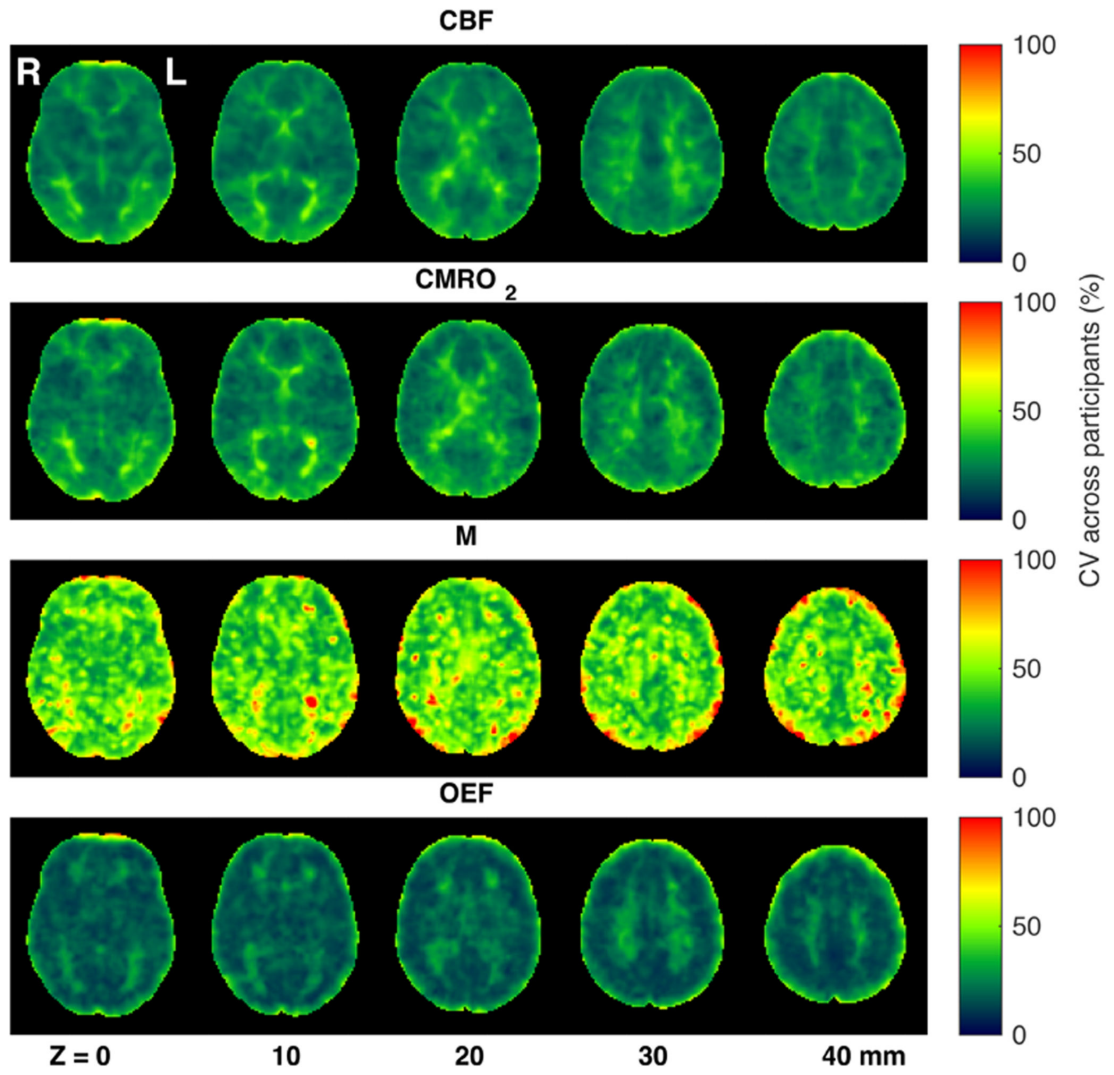
Maps of coefficient of variation (CV) across participants for each parameter acquired during rest, after registration to MNI space.

Figure 3 shows group averaged CBF_0_, OEF_0_, CMRO_2,0_, and M maps during rest. As can be seen from the figure, and consistent with expected physiology, regions of high perfusion are matched with areas of high metabolic oxygen consumption, while the oxygen extraction fraction has little variation throughout the grey matter, with increased estimates in regions with high macrovascular contamination and in white matter. Example parameter maps are also presented for a single participant in Supplementary Figure S1 and parameter maps from all participants are accessible at https://owncloud.cubric.cf.ac.uk/s/UtdmuMqU80rVRY7. Figure 4 shows maps of coefficient of variation (CV) across participants for each parameter, with the highest CV values for CBF_0_, OEF_0_ and CMRO_2,0_ appearing in white matter. Due to the long arrival times and lower perfusion rate in white matter, any parameters derived from ASL measurements in white matter are somewhat uncertain. Therefore, the apparent elevation in OEF in WM should be treated with extreme caution. However, we cannot exclude the possibility that the elevation in WM OEF is physiological in nature.

Average M maps show a significant contrast difference between grey matter and white matter, presumably because of CBV_dHb_ differences. Hotspots related to venous macrovascular contamination are also evident.

Mean GM parameter estimates at rest were 54 ± 10 ml/100g/min, 0.37 ± 0.04, 138 ± 19 μmol/100g/min, and 9.9 ± 1.5 % for CBF_0_, OEF_0_, CMRO_2,0_, and M respectively (mean ± standard deviation across subjects).

### 3.1 Accuracy of OEF measurements

To test the accuracy of the breath-hold calibrated OEF measurements, GM OEF for the rest condition was compared to a global measure of OEF in the superior sagittal sinus, using TRUST.

**Figure 5:**
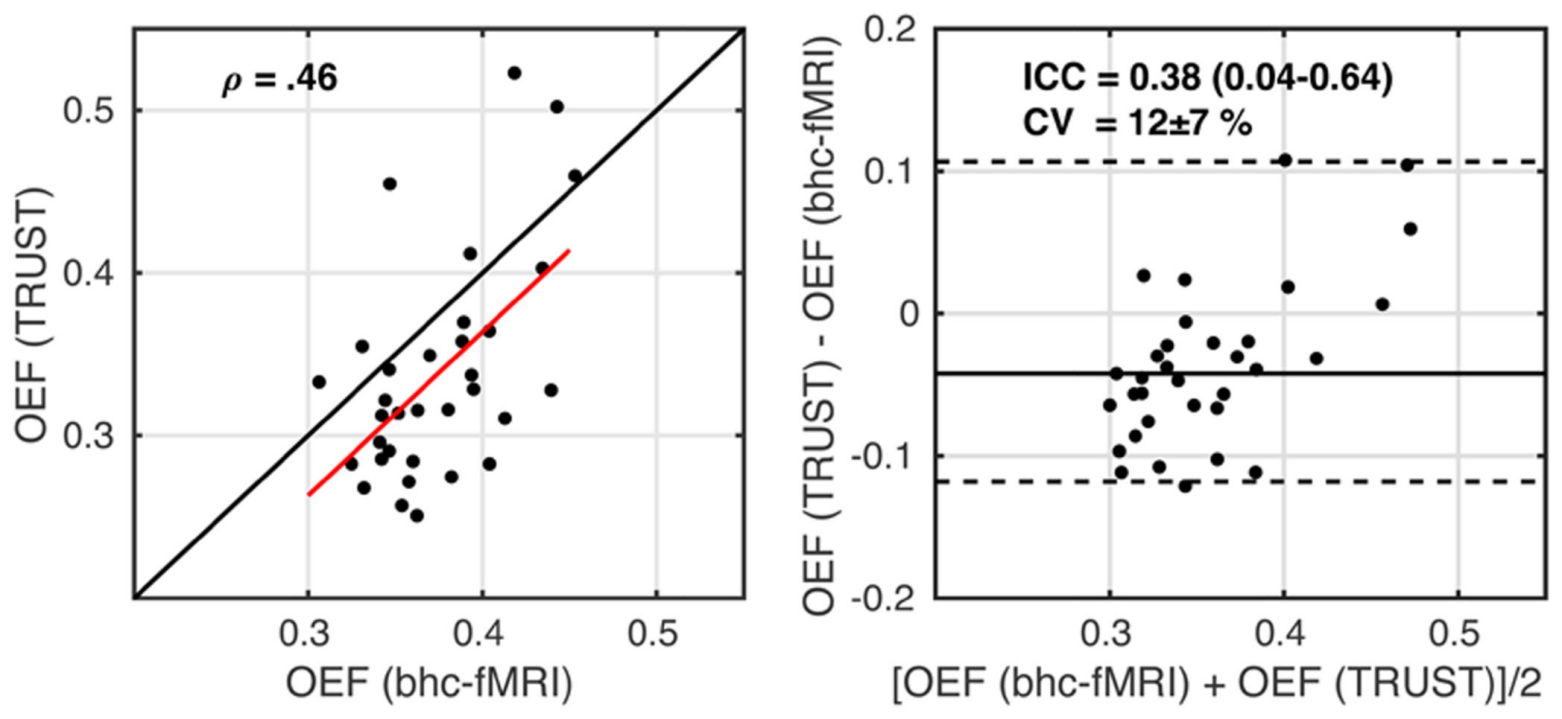
Scatterplot, left image, with the red line showing the linear regression line and the black line showing the line of unity. Bland-Altman plot, right image, comparing OEF estimates from breath-hold calibrated fMRI during rest and global OEF estimated from TRUST.

Figure 5 reports the comparison of OEF estimates between TRUST in the sagittal sinus and breath hold calibrated fMRI within the GM. Bland-Altman analysis (Figure 5, right image) demonstrated a small, non-significant apparent bias between breath-hold calibrated and TRUST estimates of OEF (0.04) with limits of agreement between -0.11 and 0.12. ICC = 0.38 (0.04-0.64 95% confidence intervals; two-way mixed effects with absolute agreement) indicates poor to fair agreement. CV was calculated between the two methods as 12 ± 7 % (mean ± standard deviation of CV across subjects). The TRUST OEF measurements showed evidence for not being normally distributed (W = 0.88; p = 0.002), so Spearman correlation coefficient between resting TRUST and breath-hold calibrated estimates was calculated as ρ(31) = .46 (p = 0.007; Figure 5, left image). For consistency with previous comparisons, the Pearson correlation was r(31) = .55 (p = 9x10^-4^), which is consistent with the performance of dual-calibrated and single gas calibration methods (Chiarelli et al., 2022).

### 3.2 Test-retest reliability

**Figure 6:**
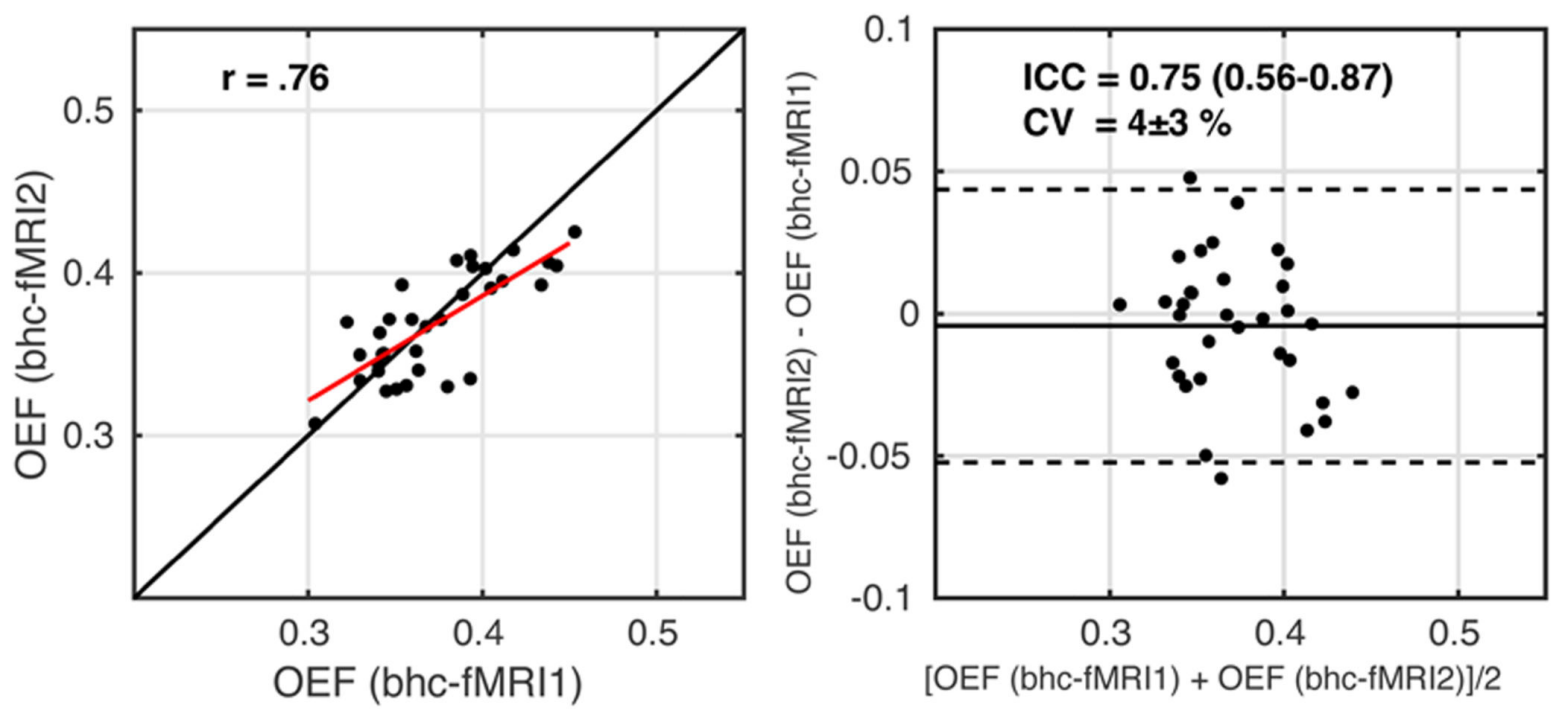
Scatterplot, left image, with the red line showing the linear regression line and the black line showing the line of unity. Bland-Altman plot, right image, comparing OEF estimates between the two measurements of breath-hold calibrated fMRI. fMRI1 – *breath hold task with grey background*; fMRI2 – *breath hold task with checkerboard background*.

Within session repeatability was assessed between the rest and visual stimulation measurements by comparing OEF averaged across GM, but excluding occipital areas to minimise between-session differences arising from the task. The GM mask was cropped by transforming the group CBF visual activation p_FWE_ map into individual space and excluding voxels with any evidence of a group level CBF response to the visual stimulation (p_FWE_<1). The resulting scatter and Bland-Altman plots are shown in Figure 6. ICC = 0.75 (0.56-0.87) indicates moderate to good reliability. CV = 4 ± 3 % between the two measurements. The correlation between within session OEF estimates during rest and visual stimulation was r(31) = .76 (p = 2x10^-7^). Regional reliability was assessed as follows. The Harvard-Oxford cortical atlas (Desikan et al., 2006), which is provided in FSL in MNI152 space, was transformed into individual space. The regional average of each parameter was calculated over voxels in the intersection of the GM mask and the Harvard-Oxford cortical regions to which a 25% partial volume threshold was applied (Craddock et al., 2012). Supplementary Table S1 reports summary OEF for each region for the two measurements, CV and ICC. The lateral occipital cortex, intracalcarine cortex, lingual gyrus, occipital fusiform gyrus and occipital pole are omitted here, as they contain areas of significant CBF response to the visual stimulus at the group level.

**Figure 7:**
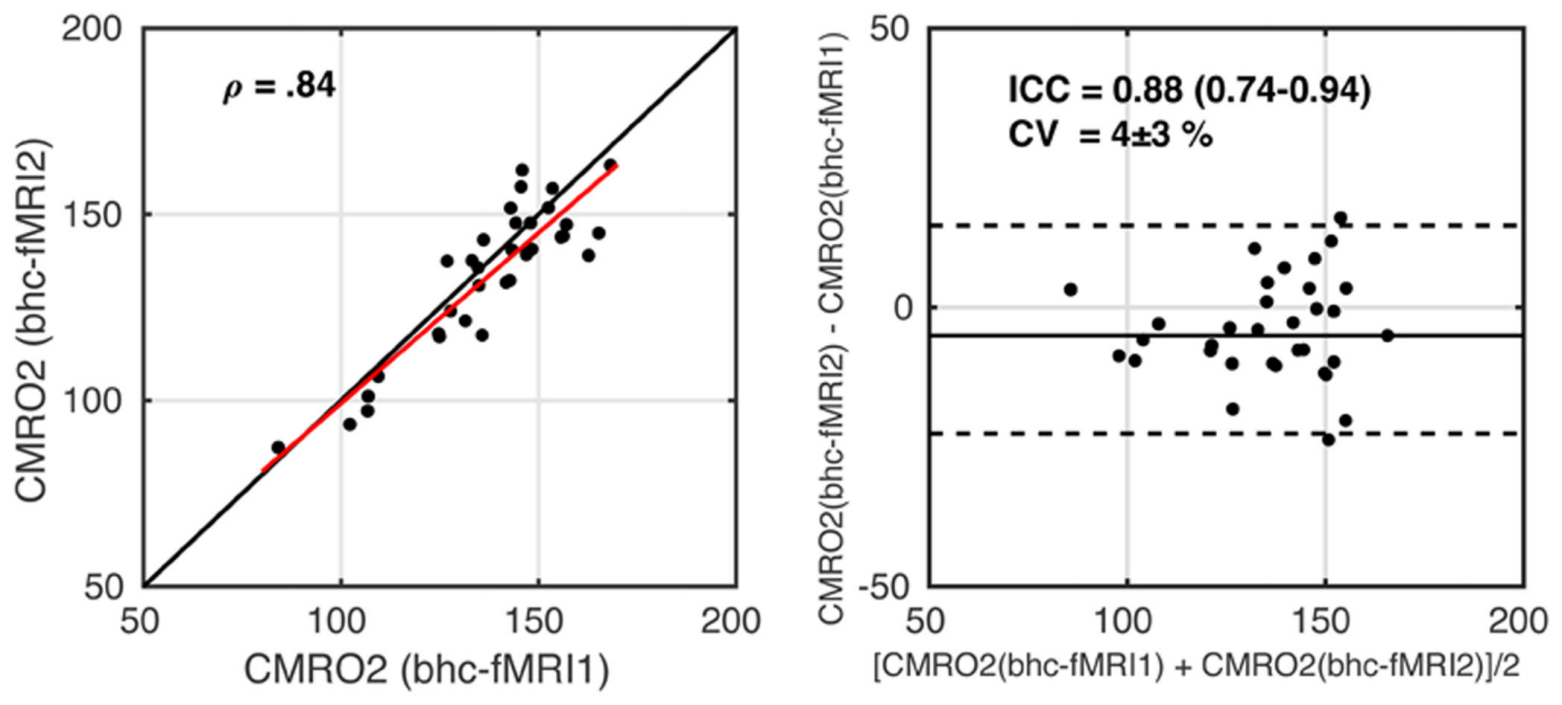
Scatterplot, left image, with the red line showing the linear regression line and the black line showing the line of unity. Bland-Altman plot, right image, comparing CMRO_2_ estimates between the two measurements of breath-hold calibrated fMRI. fMRI1 – *breath hold task with grey background*; fMRI2 – *breath hold task with checkerboard background*.

Similarly, the equivalent scatter and Bland-Altman plots for within session repeatability for CMRO_2_ are shown in Figure 7. ICC = 0.88 (0.74-0.94) indicates good to excellent reliability. CV = 4 ± 3 % between the two measurements. The correlation between within session CMRO_2_ estimates during rest and visual stimulation was ρ(31) = .84 (p = 3x10^-7^). Regional reliability is presented in Supplementary Table S2, reporting summary CMRO_2_ values for each Harvard-Oxford cortical atlas region for the two measurements, CV and ICC.

**Figure 8:**
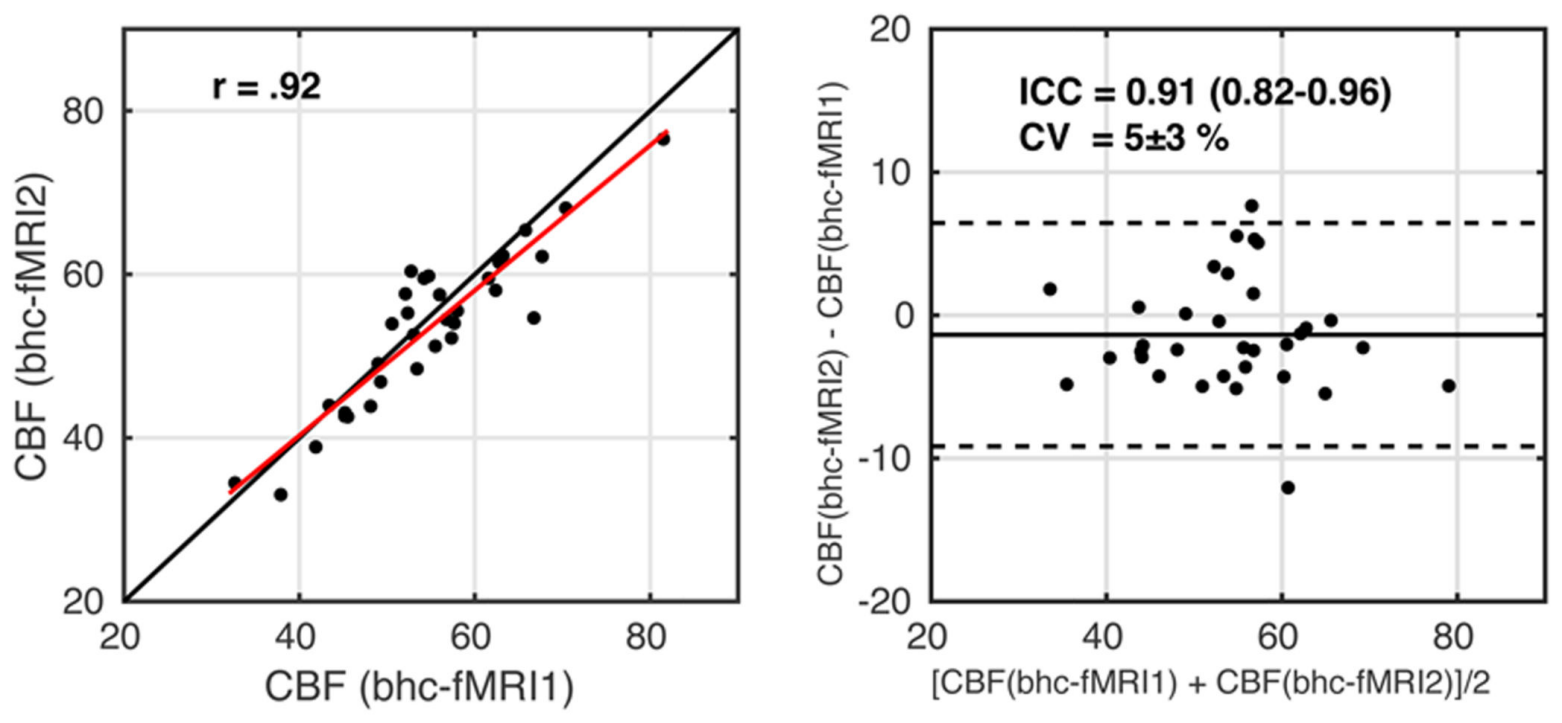
Scatterplot, left image, with the red line showing the linear regression line and the black line showing the line of unity. Bland-Altman plot, right image, comparing CBF estimates between the two measurements of breath-hold calibrated fMRI. fMRI1 – *breath hold task with grey background*; fMRI2 – *breath hold task with checkerboard background*.

For CBF, scatter and Bland-Altman plots are shown in Figure 8. ICC = 0.91 (0.82-0.96) indicates good to excellent reliability. CV = 5 ± 3 % between the two measurements. The correlation between within session CBF estimates during rest and visual stimulation was r(31) = .92 (p = 6x10^-14^).

### 3.3 Sensitivity to visual stimulation

**Figure 9:**
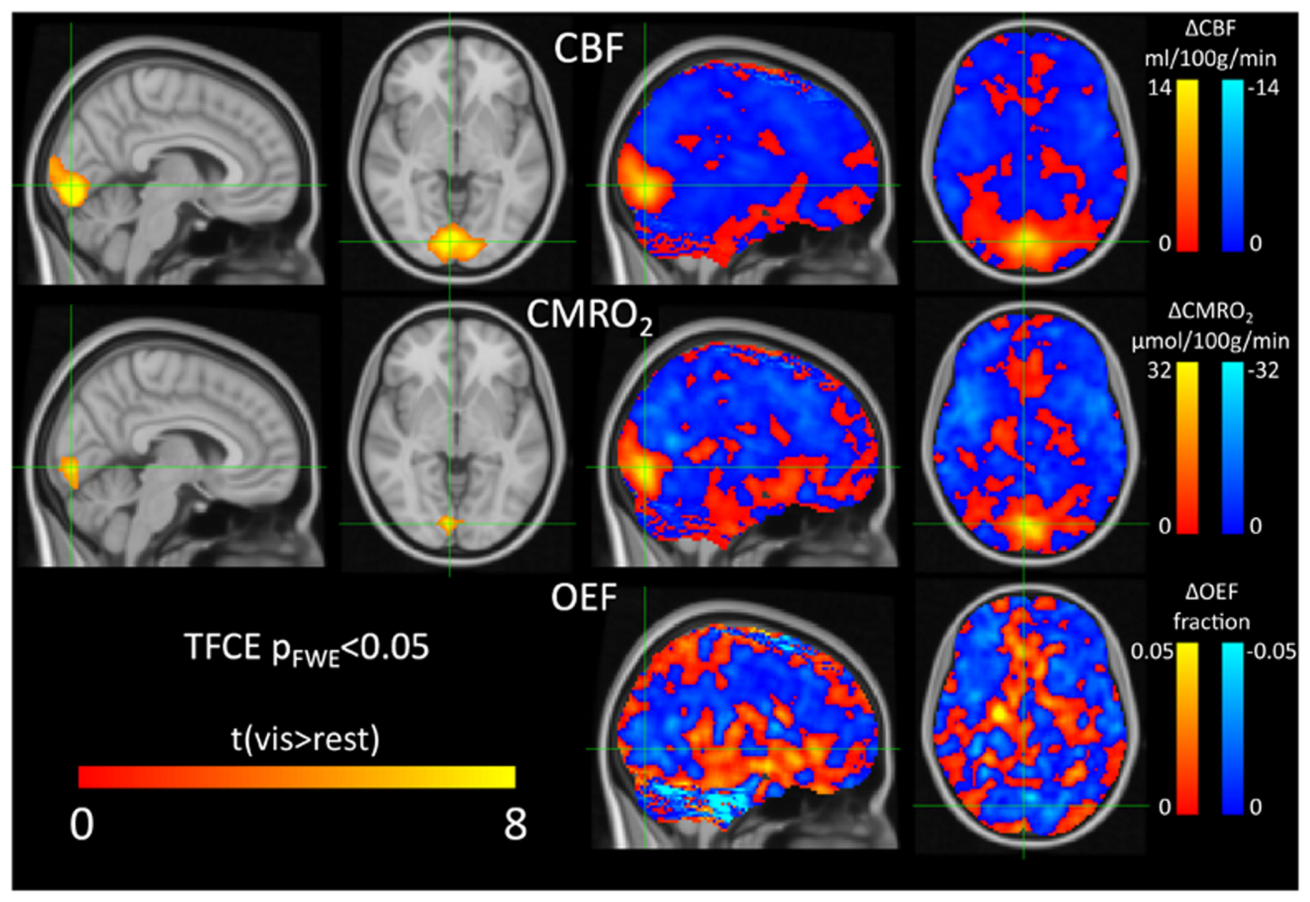
Statistical parametric group maps of CBF, CMRO_2_ and OEF changes following visual stimulations. Left side only shows t-statistic maps of significant visual response (visual condition > resting condition, p_FWE_<0.05), with OEF not shown as no regions reached significance. Right side shows maps of the change in each parameter with visual stimulation (averaged across participants).

The sensitivity of the breath-hold calibrated method to detect changes in OEF and CMRO_2_ was assessed by comparing rest and visual stimulation conditions. Figure 9 reports the voxelwise analysis of the group data in MNI space of the response to the visual task. The analysis revealed a significant increase in CBF and CMRO_2_ in the primary visual cortex associated with the task.

**Figure 10:**
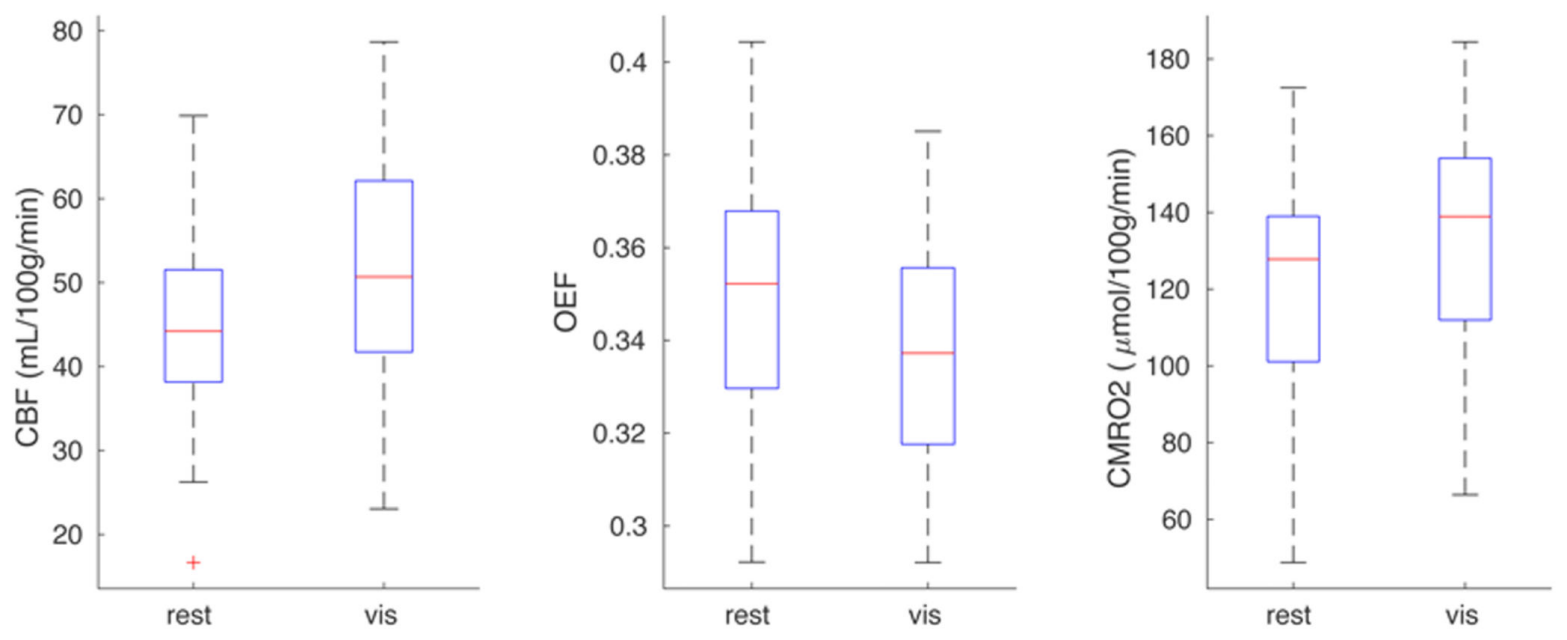
Box plots of visual ROI-averaged CBF, OEF and CMRO_2_ for rest and visual task conditions.

Figure 10 summarises the average results in an ROI defined by significant CBF visual response at the group level (p_FWE_ < 0.05). The Shapiro-Wilk test suggested no deviation from a normal distribution for either CBF (W = 0.97; p = 0.58), CMRO_2_ (W = 0.98; p = 0.81), or OEF (0.97; p = 0.59). The average CBF increase was 16 ± 2 % (mean ± SEM across subjects; p = 4x10^-9^; t(32) = 8.0) while CMRO_2_ increased by 12 ± 2 % (p = 2x10^-6^; t(32) = 5.8) within the functional ROI defined considering only significantly activated voxels. The smaller CMRO_2_ increase was associated with a mean OEF reduction of 3.3 ± 1.4 % (p = 0.013; t(32) = -2.6), with a flow-metabolism coupling constant of 1.35 consistent with recent MRI measurements during prolonged visual stimulation (Arzanforoosh et al., 2023). Flow-metabolism coupling was calculated as the %CBF increase divided by the %CMRO_2_ increase due to visual stimulation. Post-hoc power calculations for the two-tailed paired t-tests gave effect sizes of 1.0 and 0.46 and power of 1.0 and 0.73 for CMRO_2_ and OEF, respectively.

## 4 Discussion

This work presents a practical and pragmatic calibrated-fMRI method for mapping CMRO_2_ in grey matter without the need for respiratory modulation with exogenous gases. The approach is straightforward to apply and should be widely applicable to research as well as clinical studies. The method appears to have similar accuracy and precision to the gas-based methods, when compared to global measurements of OEF derived from the sagittal sinus (Chiarelli et al., 2022). In addition, it showed good repeatability when applied to different sessions of measurement.

As well as assessing the accuracy of the method we also sought to investigate the sensitivity to local changes in metabolism and physiology. To this end we repeated the measurement during rest and continuous visual stimulation, where we expected to observe the effects of a flow-metabolism coupling ratio greater than one as demonstrated by a greater increase in CBF compared to CMRO_2_ in the primary visual cortex and thus a local reduction in OEF. The reduction in OEF was statistically significant, and of a similar magnitude to that observed in other MRI measurements of prolonged visual stimulation (Arzanforoosh et al., 2023). This is consistent with the observation that the ratio of fractional increase of CBF to fractional increase of CMRO_2_ is lower during periods of extended neural activation compared to briefer stimulation, with the change in CMRO_2_ increasing with stimulation duration (Mintun et al., 2002). Therefore, the relatively small decrease in OEF observed in this study is plausible and consistent with known physiology. Nonetheless, the developed method is indeed sensitive to local changes in flow and metabolism induced by functional hyperemia.

The limitations of the method are mostly shared with the dual-calibrated methodology. For example, for the method to be viable there must be a local increase in CBF with breath-holding, which implies that there must be a local vascular reserve. This condition may not be met in diseases such as ischemic stroke, where arterial vessels may be maximally dilated in an attempt to maintain local perfusion (Krainik et al., 2005; Salinet et al., 2015; Sebok et al., 2021). The method assumes that the ratio between BOLD-sensitive and capillary blood volume as well as the permeability to oxygen of the capillary and brain tissue remains constant. Although large changes in these parameters appear unlikely, relevant modifications might occur with tissue and vascular remodelling found in brain tumors (Fierstra et al., 2016; Hsu et al., 2004; Pillai & Zaca, 2012). Additionally, the method assumes that the mean transit time through the microvasculature (MTTc) and the mitochondrial oxygen tension (PmO_2_) are not concurrently high (MTTc more than approximately 2 to 3 seconds and PmO_2_ greater than 20 to 30 mmHg) (Chiarelli et al., 2022). This scenario is assumed only to occur in cases of gross physiological and metabolic dysfunction and thus is not possible to probe with experiments in healthy volunteers. Due to these constraints, care should be taken if applying this method in stroke or other diseases where vascular remodelling and long tissue transit times may co-exist with regions of elevated PmO_2_, or where local vascular reserve may be depleted. Although still requiring validation in brain diseases that might impair its accuracy, the proposed method offers a simple means of mapping cerebral oxygen metabolism with MRI and has the potential to be a useful tool for both neuroscience research and clinical imaging.

Limitations of breath-holding in MRI have been summarised in recent reviews as participant compliance and variability in breath-hold performance (Liu et al., 2019; Urback et al., 2017; Zhao et al., 2022). However, with the bhc-fMRI measurement of CMRO_2_ and OEF, these contributions to CVR variability partly cancel due to these being present in both BOLD and CBF responses to the breath-hold, as evidenced by the lower within subject CV reported here for OEF and CMRO_2_, as compared to breath-hold CVR within subject CV measurements reported previously (Zhao et al., 2022). Recently, it has been shown that time-locked head motion is also a source of noise for breath-hold CVR measurements (Moia et al., 2020; Moia et al., 2021). One potential limitation of the analysis method employed here is the conservative mitigation for motion, relying solely on acquisition-to-acquisition registration provided by MCFLIRT (Jenkinson et al., 2002). We have taken the pragmatic approach to employ minimal motion compensation and avoid the potential pitfalls of overcorrection that can reduce the reliability of parameter estimates (Moia et al., 2021).

A potential limitation of this study is that OEF measurements are not compared with another OEF mapping method. However, due to the minimal spatial variation of OEF in the healthy brain, we prefer to make a quantitative comparison with a validated measurement of global OEF (TRUST). We also extend our previous studies with gas calibration to explore the local sensitivity to functional hyperaemia. A previous comparison of gas-calibrated OEF to superior sagittal sinus OEF (Chiarelli et al., 2022) found similar correlations between the methods (r = 0.58 for dual-gas calibration and r = 0.64 for hypercapnia-only calibration) as observed here between breath-hold calibration and superior sagittal sinus measurements of OEF.

In conclusion, we present a new MRI method for mapping CMRO_2_ and OEF without the need for manipulating inspired gas concentrations. The breath-hold approach avoids the need for a complicated gas delivery system or a face mask, which some participants find uncomfortable. This method represents an approach that may be more practical in many patient groups.

## Supplementary Material

Supplementary Material

## Data Availability

The data presented here were acquired as part of the Welsh Advanced Neuroimaging Dataset (McNabb et al., 2024). The unprocessed data is available in BIDS format from https://git.cardiff.ac.uk/cubric/wand. Processed parameter maps are available from https://owncloud.cubric.cf.ac.uk/s/UtdmuMqU80rVRY7. The visual presentation scripts for the breath-hold and breath-hold with visual stimulation tasks are available from git.cardiff.ac.uk/cubric/wand/-/tree/main/code/BreathHold. The analysis code for estimating OEF_0_ and CMRO_2_ is publicly available 10.5281/zenodo.10695877
