# Supplementary material for: Breath-hold calibrated fMRI mapping of absolute cerebral metabolic rate of oxygen metabolism (CMRO 2 ): An assessment of the accuracy and repeatability in a healthy adult population
Source: Imaging Neurosci (Camb). 2024 Sep 23;2:imag-2-00298. doi: 10.1162/imag_a_00298 (PMC7616461; doi:10.1162/imag_a_00298)
Supplement: Supplementary Material [file imag_a_00298-supp.pdf]

Supplementary Information for the Manuscript:

Breath-hold calibrated fMRI Mapping of Absolute Cerebral Metabolic Rate of Oxygen Metabolism ($CMRO_2$): An Assessment of the Accuracy and Repeatability in a Healthy Adult Population.

Contents

Page 2 - Section S1: A derivation of Equation 2

Page 5 - Figure S1: Example subject parameter maps (CBF, $CMRO_2$, M, OEF)

Page 6 - Table S1: Regional test-retest results for OEF

Page 8 - Table S2: Regional test-retest results for $CMRO_2$

S1. Maximum BOLD Signal (M) incorporating Flow-Diffusion Model of Oxygen Transport

The maximum BOLD signal change that would be obtained with a complete removal of deoxyhemoglobin from the imaging voxel (M) [1] can be approximated as:

$$M = TE \cdot A \cdot CBV_{v,0} \cdot \left(\left(1 - S_vO_{2,0}\right) \cdot [Hb]\right)^{\beta} \quad \text{(S1)}$$

where TE is the echo time of the sequence and the subscript $_0$ depicts baseline values. S_VO_2 is venous oxygen saturation and [Hb] is the concentration of hemoglobin in blood. CBV_V is the BOLD sensitive blood volume including the venous and capillary blood volumes. A and β are constants related to field strength, vessel geometry, and water diffusion in the extravascular space.

The venous oxygen saturation (S_VO_2) can be expressed as a function of the oxygen extraction fraction (OEF) and arterial oxygen content as [2]:

$$S_vO_2 = (C_aO_2 - OEF \cdot C_aO_2)/(\varphi \cdot [Hb]) \quad \text{(S2a)}$$

Or, rearranging the terms, as:

$$S_vO_2 = \frac{C_aO_2}{\varphi \cdot [Hb]} \cdot (1 - OEF) \quad \text{(S2b)}$$

where CaO_2 is the arterial oxygen concentration in blood and φ is the oxygen binding capacity of hemoglobin (fixed at φ=1.34 mlO_2/gHb).

Therefore, combining Equation S2b with Equation S1, M can be expressed as:

$$M = TE \cdot A \cdot CBV_{v,0} \cdot \left(\left(1 - \frac{C_aO_{2,0}}{\varphi \cdot [Hb]} \cdot (1 - OEF_0)\right) \cdot [Hb]\right)^{\beta} \quad \text{(S3)}$$

A compartmental model describes radial diffusion of oxygen out of a straight cylindrical capillary of unit length through the following differential equation:

$$\frac{dC_{cap}O_2(x,t)}{dt} = -k \cdot (P_{cap}O_2(x,t) - P_mO_2) \quad \text{(S4)}$$

where $C_{cap}O_2$ and $P_{cap}O_2$ are the concentration and the partial pressure of oxygen at a relative position x along and time t within the capillary, P_mO_2 is the oxygen at the end of the diffusion path, namely at the mitochondria, and k is the effective permeability of the path, combining both capillary endothelium and brain tissue [3, 4]:

Assuming steady-state, Equation S4 becomes[5]:

$$\frac{dC_{cap}O_2(x)}{dx} = -k \cdot T_{cap} \cdot (P_{cap}O_2(x) - P_mO_2) \qquad \text{(S5)}$$

where T_{cap} is the capillary transit time (CTT).

In the presence of multiple capillaries, a simplified approach neglects possible effects of CTT variability (or heterogeneity) on the net oxygen extraction of the capillary ensemble within a voxel and modifies Equation S5 by simply substituting CTT with the mean CTT (MCTT) in the capillary bed.

MCTT can be expressed as the ratio between the capillary blood volume (CBV_{cap}) and CBF obtaining:

$$CBF \cdot \frac{dC_{cap}O_2(x)}{dx} = -k \cdot CBV_{cap} \cdot (P_{cap}O_2(x) - P_mO_2) \qquad \text{(S6)}$$

Since $P_{cap}O_2$ and $C_{cap}O_2$ are quickly equilibrated (less than a few milliseconds), depending upon the Hb oxygen binding curve described by the Hill equation:

$$SO_2 = \frac{1}{1+\left(\frac{P_{50}}{PO_2}\right)^h} \qquad \text{(S7)}$$

the differential Equation S6 can be expressed as:

$$CBF \cdot \frac{dC_{cap}O_2(x)}{dx} = -k \cdot CBV_{cap} \cdot \left(P_{50} \cdot \sqrt[h]{\frac{C_{cap}O_2(x)}{\varphi \cdot [Hb] - C_{cap}O_2(x)}} - P_mO_2\right) \qquad \text{(S8)}$$

where P_{50} (mmHg) is the oxygen partial pressure when half of Hb is saturated with oxygen (generally $P_{50} \approx 26$ mmHg, h is the Hill constant, fixed at h=2.8). An explicit solution to derive OEF_0 from Equation S8 can be obtained assuming a linear decrease of $C_{cap}O_2(x)$ along the capillary, which makes the right term of Equation S8 constant, obtaining:

$$CBF \cdot \frac{dC_{cap}O_2(x)}{dx} = -k \cdot CBV_{cap} \cdot \left(P_{50} \cdot \sqrt[h]{\frac{<C_{cap}O_2(x)>}{\varphi \cdot [Hb] - <C_{cap}O_2(x)>}} - P_mO_2\right) \qquad \text{(S9)}$$

where $<C_{cap}O_2(x)>$ represents the average value of $C_{cap}O_2(x)$ in the capillary.

Assuming <$C_{cap}O_2(x)$>≈φ·[Hb]·(S_aO_2+S_vO_2)/2= φ·[Hb]·(1-OEF/2), with S_aO_2 being arterial saturation, Equation S9 can be integrated and obtain, by equating the loss of oxygen from the capillary to $CMRO_2$, the following equation:

$$CMRO_2 = CBF \cdot OEF \cdot CaO_2 = k \cdot CBV_{cap} \cdot \left(P_{50} \cdot \sqrt[h]{\frac{2}{OEF} - 1} - P_mO_2\right) \quad \text{(S10)}$$

CBV_{cap} is here assumed to be a fraction of CBV_v, i.e., CBV_v=ρ·CBV_{cap}. Substituting CBV_{cap}, from Equation S10 into Equation S3, we obtain S11, an equation for M constrained by the flow-diffusion model of oxygen exchange in the capillaries:

$$M_{diffusion} = TE \cdot \frac{A\cdot\rho}{K} \cdot \frac{OEF_0 \cdot CBF_0 \cdot C_aO_{2,0} \cdot \left(\left(1 - \frac{C_aO_{2,0}}{\varphi\cdot[Hb]}\cdot(1-OEF_0)\right)\cdot[Hb]\right)^{\beta}}{\left(P_{50}\cdot\sqrt[h]{\frac{2}{OEF_0}-1} - P_mO_{2,0}\right)} \quad \text{(S11)}$$

Supplementary References

1. Hoge RD, Atkinson J, Gill B, et al. Investigation of BOLD signal dependence on cerebral blood flow and oxygen consumption: the deoxyhemoglobin dilution model. *Magnetic resonance in medicine* 1999; 42: 849-863. Research Support, Non-U.S. Gov't 1999/11/05.
2. Merola A, Murphy K, Stone AJ, et al. Measurement of oxygen extraction fraction (OEF): An optimized BOLD signal model for use with hypercapnic and hyperoxic calibration. *NeuroImage* 2016; 129: 159-174. DOI: 10.1016/j.neuroimage.2016.01.021.
3. Germuska M, Chandler HL, Stickland RC, et al. Dual-calibrated fMRI measurement of absolute cerebral metabolic rate of oxygen consumption and effective oxygen diffusivity. *NeuroImage* 2019; 184: 717-728. DOI: 10.1016/j.neuroimage.2018.09.035.
4. Hyder F, Shulman RG and Rothman DL. A model for the regulation of cerebral oxygen delivery. *J Appl Physiol (1985)* 1998; 85: 554-564. DOI: 10.1152/jappl.1998.85.2.554.
5. Hayashi T, Watabe H, Kudomi N, et al. A theoretical model of oxygen delivery and metabolism for physiologic interpretation of quantitative cerebral blood flow and metabolic rate of oxygen. *Journal of cerebral blood flow and metabolism : official journal of the International Society of Cerebral Blood Flow and Metabolism* 2003; 23: 1314-1323. DOI: 10.1097/01.WCB.0000090506.76664.00.

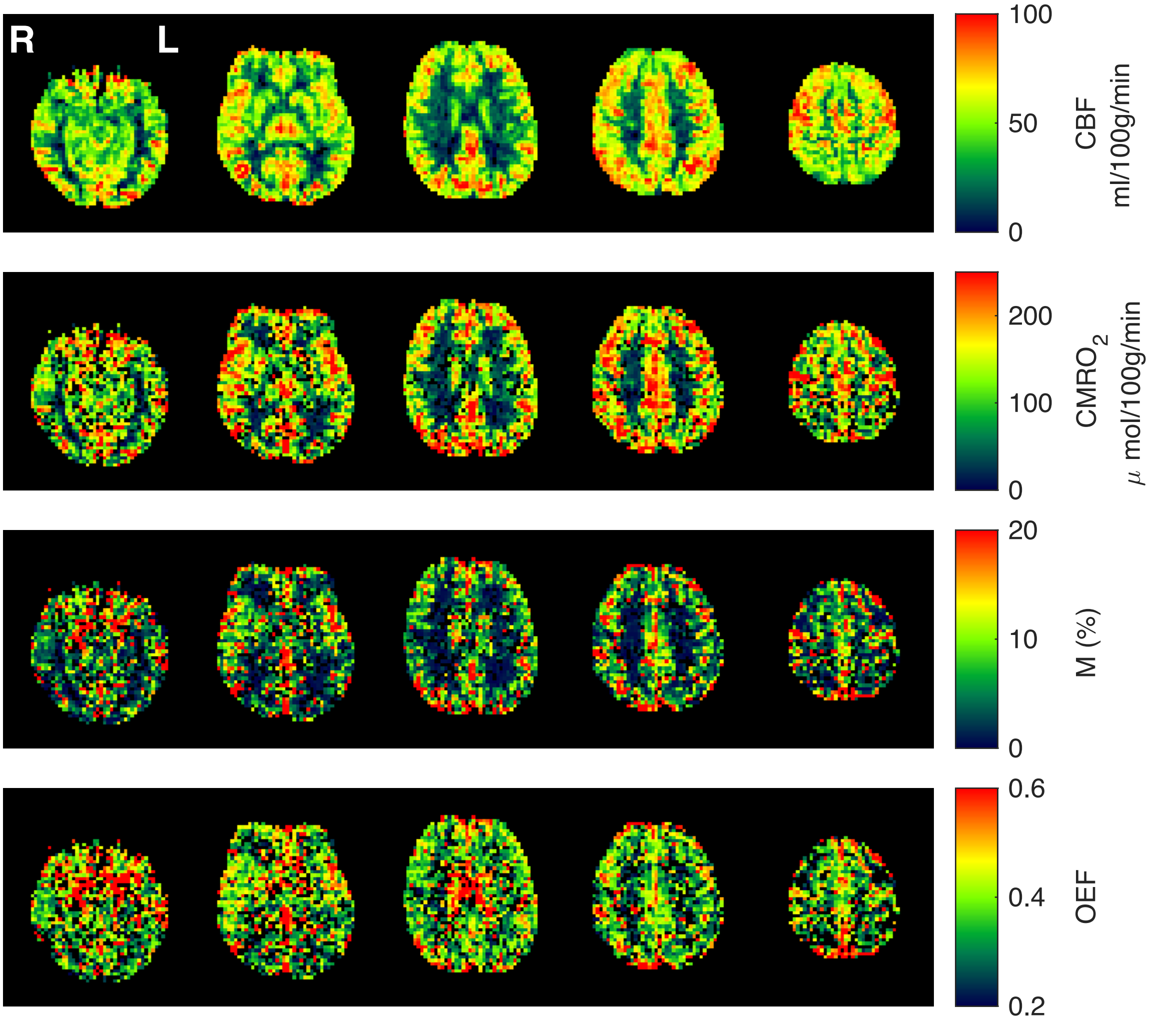

Figure S1: Example subject parameter maps

Region	OEF (1)	OEF (2)	CV (%)	ICC
Frontal Pole	0.38±0.04	0.37±0.04	5±5	0.69 (0.46-0.83)
Insular Cortex	0.35±0.05	0.35±0.04	7±4	0.60 (0.34-0.78)
Superior Frontal Gyrus	0.34±0.05	0.34±0.04	6±4	0.73 (0.52-0.86)
Middle Frontal Gyrus	0.33±0.04	0.33±0.04	5±4	0.72 (0.51-0.85)
Inferior Frontal Gyrus	0.37±0.05	0.35±0.06	6±8	0.63 (0.38-0.80)
Inferior Frontal Gyrus, pars opercularis	0.35±0.05	0.35±0.05	6±5	0.69 (0.47-0.84)
Precentral Gyrus	0.35±0.04	0.35±0.04	5±4	0.69 (0.46-0.83)
Temporal Pole	0.41±0.05	0.41±0.05	6±4	0.56 (0.27-0.75)
Superior Temporal Gyrus, anterior	0.40±0.05	0.39±0.05	8±8	0.20 (-0.15-0.50)
Superior Temporal Gyrus, posterior	0.38±0.05	0.37±0.06	8±8	0.51 (0.22-0.72)
Middle Temporal Gyrus, anterior	0.37±0.05	0.38±0.05	10±8	0.25 (-0.10-0.54)
Middle Temporal Gyrus, posterior	0.39±0.05	0.39±0.05	6±4	0.63 (0.37-0.80)
Middle Temporal Gyrus, temporooccipital part	0.37±0.05	0.37±0.05	7±5	0.60 (0.33-0.78)
Inferior Temporal Gyrus, anterior	0.39±0.08	0.38±0.08	13±11	0.12 (-0.22-0.44)
Inferior Temporal Gyrus, posterior	0.41±0.06	0.42±0.08	9±9	0.31 (-0.03-0.59)
Inferior Temporal Gyrus, temporooccipital part	0.43±0.06	0.43±0.06	6±4	0.69 (0.46-0.83)
Postcentral Gyrus	0.36±0.04	0.35±0.04	5±5	0.61 (0.35-0.78)
Superior Parietal Lobe	0.36±0.04	0.36±0.04	6±5	0.55 (0.27-0.75)
Supramarginal Gyrus, anterior division	0.35±0.04	0.35±0.04	6±5	0.58 (0.30-0.77)
Supramarginal Gyrus, posterior division	0.35±0.04	0.34±0.04	6±6	0.61 (0.34-0.78)
Angular Gyrus	0.35±0.04	0.34±0.04	5±5	0.62 (0.36-0.79)
Frontal Medial Cortex	0.44±0.07	0.43±0.07	8±6	0.60 (0.33-0.78)
Supplementary Motor Cortex	0.37±0.05	0.38±0.04	6±4	0.58 (0.30-0.77)
Subcallosal Cortex	0.41±0.07	0.43±0.08	11±8	0.38 (0.05-0.63)
Paracingulate Gyrus	0.37±0.05	0.37±0.04	5±4	0.73 (0.53-0.86)
Cingulate Gyrus, anterior division	0.37±0.06	0.37±0.04	6±4	0.73 (0.52-0.85)
Cingulate Gyrus, posterior division	0.37±0.04	0.36±0.03	5±4	0.64 (0.39-0.80)
Precuneous Cortex	0.39±0.04	0.38±0.03	4±3	0.58 (0.31-0.77)
Cuneal Cortex	0.40±0.04	0.40±0.03	4±4	0.61 (0.34-0.78)
Frontal Orbital Cortex	0.40±0.05	0.38±0.05	6±5	0.61 (0.35-0.79)
Parahippocampal Gyrus, anterior division	0.41±0.07	0.39±0.06	7±6	0.66 (0.42-0.82)
Parahippocampal Gyrus, posterior division	0.34±0.06	0.34±0.06	9±8	0.42 (0.10-0.67)
Temporal Fusiform Cortex, anterior division	0.40±0.09	0.37±0.08	15±11	0.38 (0.06-0.63)
Temporal Fusiform Cortex, posterior division	0.38±0.05	0.38±0.06	7±6	0.47 (0.16-0.70)
Temporal Occipital Fusiform Cortex	0.39±0.06	0.39±0.05	7±7	0.54 (0.25-0.74)
Frontal Operculum Cortex	0.35±0.05	0.35±0.05	7±6	0.60 (0.33-0.78)
Central Opercular Cortex	0.37±0.05	0.37±0.04	7±5	0.59 (0.31-0.77)
Parietal Operculum Cortex	0.38±0.05	0.37±0.04	6±4	0.63 (0.38-0.80)
Planum Polare	0.37±0.07	0.36±0.05	8±7	0.61 (0.34-0.78)
Heschl's Gyrus	0.37±0.06	0.37±0.05	10±7	0.35 (0.01-0.61)
Planum Temporale	0.39±0.06	0.39±0.05	8±5	0.57 (0.29-0.76)
Supracalcarine Cortex	0.38±0.05	0.37±0.05	7±5	0.56 (0.28-0.76)

Table S1: Test-retest reliability of OEF for Harvard-Oxford cortical atlas regions. Values of OEF and coefficient of variation (CV) are presented as mean ± standard deviation across participants. Intraclass correlation (ICC) is presented as ICC (95% confidence intervals), two-way mixed effects with absolute agreement. Note that a visual stimulus was presented during measurement OEF (2). The *lateral occipital cortex*, *intracalcarine cortex*, *lingual gyrus*, *occipital fusiform gyru*s and *occipital pole* are omitted here, as they contain areas of significant CBF response to the visual stimulus at the group level.

Table S2: Test-retest reliability of $CMRO_2$ for Harvard-Oxford cortical atlas regions. Values of $CMRO_2$ and coefficient of variation (CV) are presented as mean ± standard deviation across participants. Intraclass correlation (ICC) is presented as ICC (95% confidence intervals), two-way mixed effects with absolute agreement. Note that a visual stimulus was presented during measurement $CMRO_2$ (2). The *lateral occipital cortex*, *intracalcarine cortex*, *lingual gyrus*, *occipital fusiform gyru*s and *occipital pole* are omitted here, as they contain areas of significant CBF response to the visual stimulus at the group level.

Region	$CMRO_2$ (1)	$CMRO_2$ (2)	CV (%)	ICC
Frontal Pole	156±22	152±23	6±3	0.80 (0.63-0.90)
Insular Cortex	134±16	127±17	6±4	0.70 (0.36-0.86)
Superior Frontal Gyrus	152±25	152±24	6±5	0.79 (0.62-0.89)
Middle Frontal Gyrus	160±24	156±24	5±4	0.85 (0.71-0.92)
Inferior Frontal Gyrus	153±24	148±23	7±6	0.70 (0.47-0.84)
Inferior Frontal Gyrus, pars opercularis	156±22	152±22	5±4	0.79 (0.62-0.89)
Precentral Gyrus	162±22	156±21	5±4	0.77 (0.55-0.88)
Temporal Pole	140±20	138±20	7±6	0.63 (0.37-0.80)
Superior Temporal Gyrus, anterior	132±23	125±20	10±9	0.46 (0.16-0.69)
Superior Temporal Gyrus, posterior	140±20	131±23	8±6	0.62 (0.31-0.80)
Middle Temporal Gyrus, anterior	129±24	131±28	11±7	0.58 (0.30-0.77)
Middle Temporal Gyrus, posterior	147±20	144±18	6±5	0.64 (0.40-0.81)
Middle Temporal Gyrus, temporooccipital part	141±20	133±22	7±5	0.65 (0.37-0.81)
Inferior Temporal Gyrus, anterior	135±32	129±26	14±9	0.35 (0.02-0.62)
Inferior Temporal Gyrus, posterior	163±36	163±40	12±10	0.45 (0.14-0.69)
Inferior Temporal Gyrus, temporooccipital part	153±26	149±26	6±5	0.81 (0.65-0.90)
Postcentral Gyrus	154±23	150±22	5±3	0.83 (0.67-0.91)
Superior Parietal Lobe	150±26	146±25	6±5	0.79 (0.63-0.89)
Supramarginal Gyrus, anterior division	148±20	142±21	7±5	0.66 (0.41-0.81)
Supramarginal Gyrus, posterior division	148±19	141±20	6±5	0.63 (0.37-0.80)
Angular Gyrus	146±21	138±21	6±4	0.77 (0.44-0.90)
Frontal Medial Cortex	168±32	167±26	9±8	0.42 (0.10-0.66)
Supplementary Motor Cortex	176±23	173±25	5±3	0.83 (0.68-0.91)
Subcallosal Cortex	148±30	156±32	10±10	0.47 (0.17-0.70)
Paracingulate Gyrus	162±21	158±22	5±4	0.79 (0.62-0.89)
Cingulate Gyrus, anterior division	160±25	156±23	6±4	0.78 (0.59-0.88)
Cingulate Gyrus, posterior division	164±24	157±26	4±4	0.89 (0.65-0.96)
Precuneous Cortex	162±26	155±26	5±3	0.88 (0.67-0.95)
Cuneal Cortex	163±25	158±28	6±6	0.79 (0.61-0.89)
Frontal Orbital Cortex	155±23	152±25	7±6	0.70 (0.47-0.84)
Parahippocampal Gyrus, anterior division	129±24	122±17	10±6	0.52 (0.22-0.73)
Parahippocampal Gyrus, posterior division	110±21	107±22	12±8	0.54 (0.25-0.74)
Temporal Fusiform Cortex, anterior division	123±32	111±27	16±12	0.39 (0.08-0.64)
Temporal Fusiform Cortex, posterior division	124±23	121±24	8±6	0.67 (0.43-0.82)
Temporal Occipital Fusiform Cortex	133±25	131±24	7±6	0.73 (0.53-0.86)
Frontal Operculum Cortex	155±25	146±22	7±6	0.69 (0.41-0.84)
Central Opercular Cortex	150±23	145±25	5±4	0.85 (0.69-0.92)
Parietal Operculum Cortex	155±21	148±21	7±6	0.64 (0.38-0.81)
Planum Polare	145±24	134±24	9±7	0.57 (0.26-0.77)
Heschl's Gyrus	165±25	163±32	9±8	0.54 (0.24-0.74)
Planum Temporale	165±20	161±22	6±4	0.72 (0.50-0.85)
Supracalcarine Cortex	166±33	154±32	9±7	0.73 (0.44-0.87)
